# Exploring Biocontrol of Unwanted Fungi by Autochthonous *Debaryomyces hansenii* Strains Isolated from Dry Meat Products

**DOI:** 10.3390/jof8080873

**Published:** 2022-08-19

**Authors:** Helena Chacón-Navarrete, Francisco Ruiz-Pérez, Francisco J. Ruiz-Castilla, José Ramos

**Affiliations:** Department of Agricultural Chemistry, Edaphology and Microbiology, University of Córdoba, E-14071 Córdoba, Spain

**Keywords:** biocontrol, yeast, *Debaryomyces hansenii*, chemical preservatives, meat products

## Abstract

The exploration of alternatives to the use of chemical preservatives in food is a topic that has attracted great attention. The implementation of regulations associated with the reduction of these elements directly affects the production of cured meat products, with the premise of looking for more “natural” alternatives. From a previously identified collection of 24 strains of *Debaryomyces* *hansenii*, isolated from dry meat products of the “Valle de los Pedroches” (Córdoba), a screening was carried out to determine which strains had inhibitory potential against a battery of fungi belonging to the genera *Aspergillus*, *Penicillium*, and *Candida*. After a series of general trials, four strains showing the greatest potential were selected by a streak inhibition assay performed at several concentrations of NaCl. The inhibitory activity of the selected *D. hansenii* strains was later evaluated by measuring their fungal antagonistic diffusible and volatile compound production following radial inhibition and mouth-to-mouth approaches, respectively. Growth aspects, sporulation, and morphology changes were also considered during these assays. The results support ideas already raised in previous studies, such as the presence of *D. hanseniii* could imply a reduction of pathogenic fungi in food. Autochthonous yeast strains inhibited not only the mycelial growth, but also sporulation, which strengthens the biocontrol activity of this yeast. Our results show that, under certain conditions, all tested *D. hansenii* strains were able to alter the growth/development of fungi, being especially evident in the cases of *Penicillium* *expansum* and *Aspergillus* *niger*. Finally, our research can facilitate the future comparison of results in this area, since we contributed to standardize the methodology described to date, we quantified the number of yeast cells and spores used during the experiments, we homogenized growth conditions for both, yeasts, and molds, and applied an image analyzer software to quantify the growth of the studied microorganisms in solid media.

## 1. Introduction

The concern about the use of “chemical preservatives” such as NaCl or nitrites/nitrates is a growing issue within society. As a result, many measures concerning its reduction are emerging, such as the implementation of rules that regulate the amount of salt added to food [1]. During the maturation of cured meat products, colonization by molds on the surface is common and their presence may be prejudicial, since some of them have a harmful potential for the consumer. This is partly due to their ability to produce mycotoxins [2], naturally produced chemical compounds by the secondary metabolism of some fungal genera such as *Aspergillus*, *Fusarium*, and *Penicillium*, among others, whose maximum production is between 24 °C and 28 °C [3]. Along with a decreased amount of preservatives, fungal proliferation is favored, thus increasing the risk of mycotoxins consumption. *Penicillium* and *Aspergillus* genera are two of the most common contaminations causing fungi in food products [4]. It is important to state that these microorganisms are not desirable because of their possible effects on health, but, moreover, because their presence can cause undesirable odors and flavors that make the product less palatable [5]. Therefore, it is important to provide tools to the food industry to avoid or control the appearance of unwanted fungi in food.

*Debaryomyces hansenii* is a very heterogeneous species of unconventional yeast that presents osmo and halotolerant capacities [6] and has traditionally been used as a model organism in osmo and halotolerance studies in eukaryotic systems [7,8,9]. Moreover, this yeast species shows both lipolytic and proteolytic important properties [10]. *D. hansenii* inhabits a wide spectrum of niches, from seawater, oils, fruit surfaces, or cured food products such as cheeses and sausages, being the yeast with the highest presence in the latter with an average of 10^6^ CFU g^−1^ of the product [11]. Such is the versatility of *D. hansenii* that many differences between strains have been reported, including their morphology, carbohydrate metabolism, or their ability to produce toxic compounds against other microorganisms [12]. Its presence in cured food is a fundamental factor in the generation of odors and flavors thanks to its ability to produce free amino acids that act as precursors to volatile sulfur compounds such as dimethyl sulfide, dimethyl disulfide, dimethyl trisulfide, or thioacetate of thiol [13].

In general, the use of yeasts as biocontrol agents against pathogenic fungi is a more than feasible alternative thanks to the intrinsic characteristics such as fast growth rates, simple nutritional requirements, or their ability to colonize a multitude of niches to compete for nutrients and space [14,15]. All of these, added to *D. hansenii*’s specific characteristics, are what make this yeast one of the biological agents recommended by the European Food Safety Authority [16]. The use of *D. hansenii* is, therefore, an especially interesting alternative in the agri-food industry.

Several reports have focused on the mechanisms involved in the inhibitory capacity of *D. hansenii* against unwanted microorganisms, but the information available is very heterogeneous and even contradictory. Nutrient competition has been proposed to be mainly involved in the inhibition of unwanted molds [17,18]. However, there are also clear indications that this yeast produces several metabolites, volatile and soluble, which could have an impact on its fungal inhibitory activity [19]. Other mechanisms are linked to the high adhesion capacity of this yeast, its ability to form biofilms, or the secretion of specific enzymes [20].

From a previously identified collection of *D. hansenii* strains, isolated from the surface of classical Spanish dry fermented pork meat products “salchichón”, “chorizo”, and “lomo ibérico” [10], we aimed to select those strains with greater potential as biocontrol agents against pathogenic fungi. 

## 2. Materials and Methods

### 2.1. Strains and Culture Conditions

The *D. hansenii* strains used in this study were taken from a previous collection of yeasts isolated from Valle de los Pedroches Iberian dry meat products [10]. Moreover, the laboratory wild-type strain CBS767 (Fungal Biodiversity Center, The Netherlands) [8] was used as a comparative control. To carry out the different tests, four species of potentially pathogenic fungi were used: *Aspergillus niger* HCH1 (Laboratory collection, University of Córdoba, Córdoba, Spain), *Penicillium expansum* PX (Instituto de la carne y productos cárnicos, University of Extremadura, Cáceres, Spain), *Penicillium verrucosum* PV21 (Instituto de la carne y productos cárnicos, University of Extremadura, Cáceres, Spain), and *Candida albicans* 12C (Broad Institute of MIT and Harvard, Cambridge, Massachusetts, USA) [3,21].

Yeasts were grown on YPD media (YPDA) containing 1% yeast extract, 2% peptone, and 2% dextrose (D-glucose) (*w*/*v*). Solid media was prepared by adding 2% (*w*/*v*) agar. Molds were grown on commercial PDB (Potato Dextrose Broth) (24 g/L Scharlau, Barcelona, Spain) or PDA (Potato Dextrose Agar) (39 g/L OXOID, Basingstoke, Hampshire, UK). Both yeasts and molds were incubated at 28 °C on solid media for two and five days, respectively. Subsequently, they were sealed with parafilm and kept at 4 °C. Yeasts and molds were refreshed every week and two weeks, respectively.

### 2.2. Primary Screening: Pathogenic Fungi Colonization Prevention Assay with Biofilms from D. hansenii Strains

The assay was based on the described ability of *D. hansenii* to create biofilms capable of avoiding the colonization of other microorganisms [20]. A preculture of the different strains of *D. hansenii* were grown in YPD broth at 28 °C with an agitation of 150 rpm. *D. hansenii* cell suspensions (150 μL, 1 A_600_) were spread on YPDA plates and incubated at 28 °C for 24 h. To obtain the spores, each of the different molds was inoculated in 250 mL of PDB medium. *A. niger*, *P. verrucosum*, and *P. expansum* were incubated for four days at 28 °C with constant agitation at 150 rpm. When recollecting the molds in the flasks, they had formed mycelium in the form of spheres, which were discarded. An amount of 5 μL of supernatant were inoculated in the center of each plate. In the case of *C. albicans*, cells were obtained in the same way as described for *D. hansenii* and, again, 5 μL of cell suspension were inoculated in the center of each plate. The cocultured plates were incubated for 72 h at 28 °C. Three independent biological replicates were performed. If no fungus growth was observed, the results were ranked as positive. If it was proliferation, the results were taken as negative.

A similar complementary study was performed. On petri plates with PDA, 150 μL of spore suspension of the molds or cell suspension from *C. albicans* were inoculated. Immediately after this step, drops of 5 μL of different *D. hansenii* strain cell suspensions were inoculated on each plate. The plates were incubated for 72 h at 28 °C, as described in the main assay.

### 2.3. Biotyping

MALDI-TOF MS was performed as a complementary approach to conventional identification previously published by Ramos et al. [10]. The isolates were subcultured on YPDA and grown at 28 °C. After incubation, 10 mg of cells were collected, suspended in 300 μL of sterile distilled water, and mixed thoroughly. An amount of 900 μL of absolute ethanol was added, and the samples were treated according to the method reported by Van Veen et al. [22]. Each isolate was analyzed in duplicate. For each isolate, the highest score of a match against a spectrum in the database was used for identification. The meaning of the score values were: a score between 0.00 and 1.69 was considered not to have generated a reliable identification; a score between 1.70 and 1.99 was considered identification to genera, and a score of between 2.00 and 3.00 was used for species identification [22].

### 2.4. Secondary Screening: Streak Inhibition Assay

After a first screening of the inhibitory potential of 24 *D. hansenii* strains against *A. niger*, *P. verrucosum*, *P. expansum*, and *C. albicans*, the number of *D. hansenii* strains to be further studied was reduced. The purpose of the assays described below was to verify whether the selected yeast strains possessed inhibitory potential concerning the mechanisms described for nutrient competition [17,18] as well as for diffusible and volatile inhibitory compounds production [19].

One half of the YPDA plate was spread with the yeast cell suspension of each strain. The half-cultured plates with *D. hansenii* strains were incubated at 28 °C for 48 h. With a single sampling with an inoculation loop, three consecutive streaks of fungus were made on the remaining half of each plate, starting next to the already grown yeast, without touching it. This process was applied to each combination of the five *D. hansenii* strains and fungus. All combinations were performed in triplicate. The plates were incubated at 28 °C for five additional days. This experiment was carried out under three different conditions: standard, with 0.5 M of NaCl, and with 1 M of NaCl. The inhibitory potential was calculated as follows: IP (%) = [(C − T)/C] 100, where C (control) was the maximum width of the control fungal streaks in the absence of *D. hansenii*, and T (treatment) was the maximum width of fungal streaks in the cocultured plates.

### 2.5. Radial Inhibition Assay on Solid Media

The antagonistic potential of *D. hansenii* to inhibit the fungal growth of the four strains of pathogenic fungi was performed by the agar plate inhibition assay as described by Medina-Córdova et al. [20] with a minor modification: a disk of 5 mm of sterile paper was used as a predetermined starting growth area. 

*D. hansenii* cell suspensions (10^8^ cells/mL) of the different strains were prepared by growing the yeasts on YPDA plates. The cells were counted with a Neubauer chamber (40× magnification). 

To obtain spores, at least five PDA plates per mold were inoculated and incubated for five days at 28 °C. With a Digralsky handle and sterile distilled water, the spores were collected in Falcon tubes. Spore suspensions were passed through sterile filters (Monodur NYLON-10) to remove the mycelium. Spore counting was also performed with a Neubauer chamber (40× magnification) and adjusted to 10^6^ spores/mL.

One hundred microliters of *D. hansenii* cell suspensions were spread on PDA plates, and 10 μL of mold spore suspension were inoculated in the center of each plate on a 5 mm diameter sterile paper disk. After seven days of incubation at 28 °C, the fungal growth was determined by measuring the diameter of each colony. The radial inhibition (RI) was calculated as follows: RI (%) = [(C − T)/C] 100, where C (control) was the average diameter of fungal colonies in the absence of *D. hansenii*, and T (treatment) was the average diameter of fungal colonies on the cocultured plates. For *C. albicans* replicas, the same procedure was applied but the YPDA plates were used instead of the PDA. 

When collecting the results, samples were visualized in plain view and under the microscope to study if there were morphological changes.

### 2.6. Volatile Compounds Inhibition Assay 

The effect of volatile compounds generated by *D. hansenii* strains against potential pathogenic fungi was assessed using the ‘‘mouth-to-mouth” method [20]. The yeasts and molds were prepared as described in the previous section. In one plate of PDA, 100 μL of *D. hansenii* cell suspension was spread, while in another one, 10 μL of mold spore suspension or *C. albicans* cell suspension was dropped on a 5 mm diameter sterile paper disk. Plates containing *D. hansenii* and the unwanted fungi were confronted, sealed with parafilm, and incubated at 28 °C for seven days. Plates without *D. hansenii* inoculum were used as controls. Inhibition was determined by measuring the mold colony diameter on day seven. The inhibitory activity was expressed as described in Section 2.5.

When collecting the results, samples were visualized in plain view and under the microscope to study if there were morphological changes.

### 2.7. Data Quantification and Statistical Analysis

All experiments were repeated at least three times and three technical replicates for each sample were performed. In some experiments, results were quantified using the software Image J (v.1.53) (open access), the data were analyzed with Microsoft Excel 2016 (Licensed by University of Córdoba, Microsoft Corporation, Redmond, Washington, USA) software and the significance of differences between mean values was determined by GraphPad Prism 7 (Licensed by University of Córdoba, Dotmatics, Boston, Massachusetts, USA). Significant differences are indicated with asterisks (* *p* < 0.05, ** *p* < 0.01, *** *p* < 0.001). 

## 3. Results

### 3.1. Primary Screening

The first screening of our collection strains indicated that, in most cases, the presence of *D. hansenii* prevented the proliferation of fungi, as shown in Table 1. The proliferation was sometimes observed but in a very scarce way, knowing that we made the described punctuation system to differentiate between yeast strains’ actions to choose the ones with greater apparent potential, which is the objective described for the primary screening.

In the complementary approach tested by inoculating the spores before the inoculation of yeasts, difficulties were encountered. When collecting the results, the fact that different *D. hansenii* strains were inoculated in each plate made it impossible to identify with certainty which strains were responsible for the observed changes. Based on the results of the study on the ability of *D. hansenii* to prevent the colonization of molds through biofilms, the strains CBS767 (laboratory control), LR2, LRC1, LRC2, and SRF1 of *D. hansenii* were selected to further study.

### 3.2. Biotyping

The selected *D. hansenii* strains mentioned above (CBS767, LR2, LRC1, LRC2, and SRF1) were previously identified by more conventional methods [10]. We now applied the biotyping technique MALDI-TOF, allowing us to measure values higher than two in all cases, thus confirming that all strains tested belonged to *D. hansenii* (Table S1). 

### 3.3. Secondary Screening: Streak Inhibition Assay

Figure S1 is a representative example of the streak inhibition assay previously described in Section 2.4. The image on the left shows the growth of *P. verrucosum* on YPDA without *D. hansenii*, and, on the right, streaks of the same fungus on YPDA with *D. hansenii* LRC1. The images show a noticeable difference between the control plate with no yeast and the treatment one. A gradient of inhibition from the zone closest to the yeast in the cocultured plate to the furthest is also evident. We can also observe that the maximum growth of each of the fungi streaks in the cocultured plates is still lower than the control plates. This was noted in all replicas. This fact makes us believe that not only competition for the nutrient mechanism is present in the inhibition, but also the production of some metabolite with an antagonistic effect to the fungal growth. With the positive results obtained for the standard assay, two more conditions were studied with the addition of 0.5 M NaCl and 1 M NaCl. 

**Figure 1:**
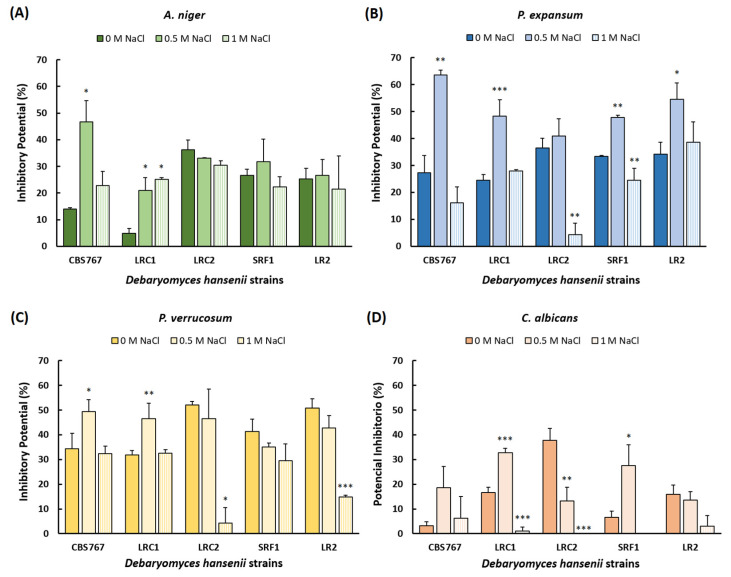
Streak inhibition assay in different microorganisms. (**A**–**D**) Inhibitory potential (%) of the different strains of *D. hansenii* in *A. niger* (**A**), *P. expansum* (**B**), *P. verrucosum* (**C**), and *C. albicans* (**D**). Mean values ± standard deviation obtained in three independent experiments are plotted. Statistical significance shown in the figure (* *p* < 0.05, ** *p* < 0.01, *** *p* < 0.001) represents the most significant data for inhibitory potential values in each selected strain of *D. hansenii* at different NaCl concentrations (0.5 M and 1 M) compared to the standard (0 M). More detailed statistical analysis can be found in the supplementary data (Tables S2 and S3).

Results in Figure 1A–D show that, in most cases, inhibition was present, with certain differences between strains and conditions. We observed how the inhibitory potential (IP) of the different strains was affected by the salt concentration of the medium. Another general observation throughout the experiment is that, under conditions of the highest concentration of NaCl studied, the inhibitory effect was reduced. The most relevant data found for *A. niger* inhibition was that the strain with the highest inhibitory potential was LRC2 under standard conditions and CBS767 at 0.5 M NaCl (Figure 1A). No mentionable differences were observed between the inhibitory potential at different NaCl concentrations. For *P. expansum*, the strain with the highest inhibitory potential was LRC2 under standard conditions, and very close to CBS767 and LR2 at 0.5 M NaCl (Figure 1B). Important differences were observed between the action of the different strains under conditions of increased salt concentration. For *P. verrucosum*, the greatest inhibitory potential was observed with LRC2 and LR2 under standard conditions. The inhibitory potential against this fungus was positively affected by increased salt (0.5 M NaCl) in CBS767 and LRC1 strains, while in the rest of strains, it was negatively affected (Figure 1C). Finally, as for *C. albicans*, the data obtained for the different treatments were very heterogeneous, where the inhibitory potential was favored by 0.5 M NaCl in some of the strains, but the greatest inhibition was observed in the case of LRC2 strain and under standard conditions (Figure 1D). The statistical significance of the data appears in Tables S2 and S3.

### 3.4. Radial Inhibition Assay 

Notable differences were observed both in the type of growth and spore production of the fungi in the presence of the *D. hansenii* strains (Figure S2). Inhibition of the fungi was observed in all cases when the radial inhibition assay was performed. A good example in Figure S2 is the case of *A. niger*, since all the *D. hansenii* strains affected the colony pigmentation due to the reduced number of spores produced by the mold. Interestingly, and in contrast to the results shown in the streak inhibition assay, we observed a greater negative influence on the growth of *C. albicans* in the presence of all *D. hansenii* studied. 

**Figure 2:**
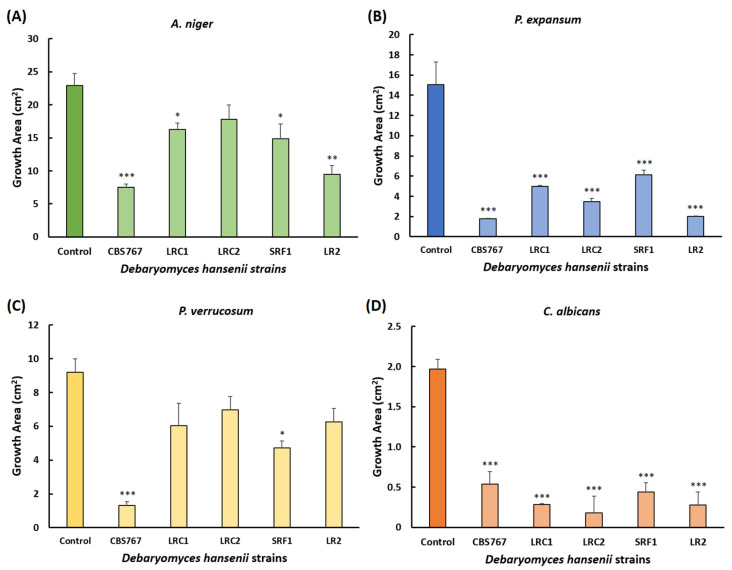
Radial inhibition of potentially pathogenic fungi by selected strains of *D. hansenii.* (**A**–**D**) Growth area (cm^2^) of the different fungi *A. niger* (**A**), *P. expansum* (**B**), *P. verrucosum* (**C**), and *C. albicans* (**D**). Mean values ± standard deviation obtained in three independent biological replicates are plotted. Statistical significance shown in the figure (* *p* < 0.05, ** *p* < 0.01, *** *p* < 0.001) represents the significant influence of the different *D. hansenii* strains on the mycelial growth of the different fungus with respect to the control plates. More detailed statistical analysis can be found in the supplementary data (Table S4).

Figure 2A–D shows how the growth area (measured with Image J) of the different fungi was affected by the presence of *D. hansenii*. The strain that showed the highest inhibitory efficiency was CBS767, whose inhibitory activity (IA) always exceeded 60% and, compared to control plates, the maximum growth never exceeded 32% (not shown).

### 3.5. Volatile Compounds Inhibition Assay

In this experiment, the data concerning IA (inhibitory activity) varied between replicates, and therefore this could not be reliably quantified. However, as mentioned in the previous section, there was a noticeable decrease in the apparent spore production, which was especially relevant in the case of *A. niger*, since the absence of sporulation was observed in all replicates (Figure S3). 

**Figure 3:**
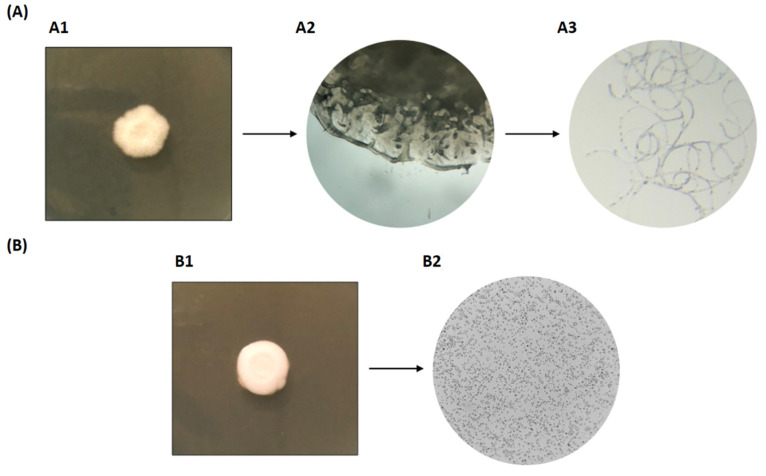
Representative images of the action of volatile compounds produced by the LRC1 strain against *C. albicans*. (**A**) *C. albicans* after incubation together with LRC1, as described in Section 2.6. (**A1**): Image of a colony, (**A2**): microscopic image of the structure of the colony (40× magnification), (**A3**): microscopic image of a scraped sample of the *C. albicans* colony structure (40× magnification). (**B**) Control without *D. hansenii* yeast. (**B1**): Image of a colony, (**B2**): microscopic image of cells in the colony (40× magnification).

In addition, and although no noticeable changes in macroscopic growth were detected for *C. albicans*, a remarkable differential result was observed in the morphology of that yeast in the treated plates when compared to the control. When checking the treated plates with any of the *D. hansenii* strains, *C. albicans* had developed a mycelial disc with different aspect and structure in comparison to the control where the normal aggregated cell colony was formed (Figure 3). 

## 4. Discussion

The use of *D. hansenii* as biocontrol agent in the food industry has been widely proposed and accepted, although there is a wide range of results in the different studies to date that indicates strain differences and various modes of action [18,19,20,23,24]. Using a *D. hansenii* autochthonous strain collection isolated from Iberic cured meat products from “Valle de los Pedroches”, we analyzed its inhibitory capability against four unwanted and potentially pathogenic fungi species in the food industry. In the present work, we showed that, under certain conditions, all the strains studied have the sought activity. Previous studies have proposed the existence of diverse mechanisms involved in the biocontrol activity exerted by *D. hansenii* [17,18,19,20]. Therefore, based on the results from the primary screening, we selected four strains to obtain further details on those proposed mechanisms. That first approach showed that the prior presence of this yeast on solid media prevents its contamination with pathogenic fungi, a fact that might have a special interest in further studies. A second screening was performed with the four strains selected (LR2, LRC1, LRC2, and SRF1). We included different NaCl concentrations in our experiments because it was reported that salt concentration affects the metabolism of *D. hansenii* [25].

In the streak inhibition assay, we observed that there is a clear inhibition gradient from the area closest to the yeast to the furthest (Figure S1). Moreover, it is clearly visible that the maximum growth of the fungi was lower in the cocultured plates (Figure S1 right) compared to the control without the *D. hansenii* selected strains (Figure S1 left). This makes us think that, in addition to the competition and consumption of resources produced by the presence of the yeast, there should also be some other elements such as volatile compounds, diffusible compounds, or even the production of toxic proteins that affect inhibition as previously proposed [17,19,20,26]. Within our results, we generally see that even with global positive results, there are differences between the inhibitory efficiency of the different strains against the different fungi. Results reported by Medina-Córdova et al. [20] indicated that inhibitory activity observed during radial diffusion assays ranged between 97 and 93%, while our results for the equivalent test were between 22 and 86%. This wide range in the quantification of the results may be explained on the basis of the different strains of *D. hansenii* used as well as the pathogenic fungi studied. Altogether, our results confirm that, although these experiments are a good initial approximation, specific individualized studies with each fungus are needed prior to yeast application [23]. In our radial diffusion assay, the maximum inhibitory activity occurred for LRC2 against *C. albicans*, a fact that is particularly striking since in the rest of the assays’ inhibition was absent or very low. We also found very little information related to the inhibitory action of *D. hansenii* against *C. albicans* and, although in those studies different conditions were used, the authors also reported antagonistic activities against *C. albicans* [12,27]. In fact, these studies propose the existence of a *D. hansenii* mycocin [12] and a killer protein [27] with inhibitory activity against that pathogenic yeast. For the rest of the fungi studied, the maximum inhibitory activity observed was with the use of CBS767, with values of 67% against *A. niger*, 88% against *P. expansum*, and 84% against *P. verrucosum*.

The production of volatile compounds is an important antagonistic mechanism used by yeast against other fungi, with its main action is usually related to their absorption through fungal cell membranes, affecting its permeability and ion exchange capacity, with the consequent loss of homeostasis [20,26]. The volatile compound inhibition assay described by Medina-Córdova et al. [20] reported the growth of fungal isolates of *Fusarium proliferatum* and *F. subglutinans* were inhibited by *D. hansenii* volatile compounds by 54.2 and 43.5%, respectively. We have used conditions similar to those reported in that work but, perhaps due to the different fungi studied, our study did not show any statistically significant effect on fungal growth. However, what we observed is a noticeable decrease or even an absence of apparent sporulation in the treated plates. This fact has been previously mentioned in other studies, but as far as we know, and in relation to the use of *D. hansenii*, neither quantitative data nor images supporting the proposal were provided [20,26,28,29].

We have not yet identified which specific volatile compounds are responsible for the observed effects. However, reviewing one of the previous studies on which this trial is based [19], we found the authors identified up to 51 volatile compounds produced by *D. hansenii* using GC-MS (gas chromatography coupled to mass spectrometry). Moreover, in the presence of *P. verrucosum*, one of the fungi used in our work, only three compounds were constantly detected in all replicates. Those compounds were 2-methyl-1-propanol, 3-methyl-1-butanol, and 2-methyl-1-butanol; all compounds associated with the appearance of flavors and odors characteristic of cured meat products [5]. A more recent paper confirmed the inhibitory activity of these compounds in addition to adding new ones to the list: 3-methylbutanoic acid, acetone, 2-phenylethanol, and 2-pen-tanone. The latter are specifically associated with inhibition of germination and mycelial growth of *Candida* and *Penicillium* species. [29]. To our knowledge, our research is the first report that specifically shows images of how the diffusible and volatile compounds’ action affects sporulation.

Another interesting precedent related to inhibitory mechanisms is the hypothesis of a killer protein produced within the diffusible compounds of *D. hansenii* when it is confronted with other fungi. Çorbacı et al. [30] reported a molecular mass of a killer toxin of about 31.5 kDa, with its highest stability at pHs between 2.5 and 5.5 and up to 37 °C. On the other hand, in Al-Qaysi et al. [27], an apparently different killer toxin was purified. That protein was reported to be around 22 kDa, with its highest stability at pH 4.5 and 25 °C. On the basis of these results, we cannot discard the coexistence of more than one toxic protein produced by *D. hansenii*, but it is crucial to keep in mind that, again, the strains and conditions used were different, and therefore much more research in the area is needed.

## 5. Conclusions

Our results reinforce the idea that *D. hansenii* could be an excellent option as a biocontrol agent. We showed that our selected *D. hansenii* strains have antagonistic activities against undesirable fungi under laboratory conditions. Based on our results, we support the idea that the antagonistic activities of the *D. hansenii* strains affect both mycelial growth and spore formation and are, most probably, diverse, including competition and the production of different antifungal metabolites.

Considering the heterogeneity of conditions used in the different studies until now and knowing that the conditions used were prone to obtain results, we propose the concentration of yeasts and spores used in the present work, as well as the use of an image analyzer software, as standard protocols to be used in further studies. Future research should include killer proteins, volatile and diffusible compound identification and scaling tests for biocontrol industrial application.

## Figures and Tables

**Table 1:** Representation of the results obtained in the study of pathogenic fungi colonization prevention assay with biofilms from *D. hansenii* strains (primary screening).

Isolation Origin	*Debaryomyces hansenii* Strains	*Aspergillus niger*	*Penicillium* *expansum*	*Penicillium* *verrucosum*	*Candida albicans*
Laboratory control	CBS767	+ + +	+ + +	+ + +	+ + +
“Chorizo Ibérico”	CB1	+ + +	+ + +	+ + +	+ + +
	CB2	+	+ + +	+ + +	+ + +
	CRC1	+	+ + +	+ + +	+ + +
	CRC2	+	+ + +	+ + +	+ + +
	CRF1	+ +	+ + +	+ + +	+ + +
	CRF2	+ +	+ + +	+ + +	+ + +
	CRF3	+	+	+	+
“Lomo Ibérico”	LB1	+ + +	+ + +	+ + +	+ + +
	LB2	+ + +	+ + +	+ + +	+ + +
	LB3	+	+ + +	+ + +	+ + +
	LR1	+ + +	+ + +	+ + +	+ + +
	LR2	+ + +	+ + +	+ + +	+ + +
	LRB1	+ + +	+ + +	+ + +	+ + +
	LRB2	+ + +	+ + +	+ + +	+ + +
	LRB3	+ + +	+ + +	+ + +	+ + +
	LRB4	+ + +	+ + +	+ + +	+ + +
	LRC1	+ + +	+ + +	+ + +	+ + +
	LRC2	+ + +	+ + +	+ + +	+ + +
	LRF1	+ +	+ + +	+ + +	+ + +
	LRF2	+ + +	+ + +	+	+
“Salchichón Ibérico”	SRC1	+ + +	+ + +	+ + +	+ + +
	SRC2	+ + +	+ + +	+ + +	+ + +
	SRF1	+ + +	+ + +	+ + +	+ + +

The results were taken as positive or negative in such a way that “ + + + “ indicates the absence of growth of the fungi under study in the three independent biological replicates; “+ + “ indicates residual growth in only one of the three independent biological replicates; “+” indicates fungal growth at least in two of the three replicates; “-” indicates that normal fungal growth similar to the control in the absence of *D. hansenii* was observed in the three independent biological replicates.

## Data Availability

The data supporting the findings of this work are available within the paper and its supplementary files.

## Supplementary Materials

The following supporting information can be downloaded at: https://www.mdpi.com/article/10.3390/jof8080873/s1.
