# Supplementary material for: Exploring Biocontrol of Unwanted Fungi by Autochthonous Debaryomyces hansenii Strains Isolated from Dry Meat Products
Source: J Fungi (Basel). 2022 Aug 19;8(8):873. doi: 10.3390/jof8080873 (PMC9410373; doi:10.3390/jof8080873)
Supplement: Supplementary file 1 [file jof-08-00873-s001.zip › jof-1828367-supplementary.pdf]

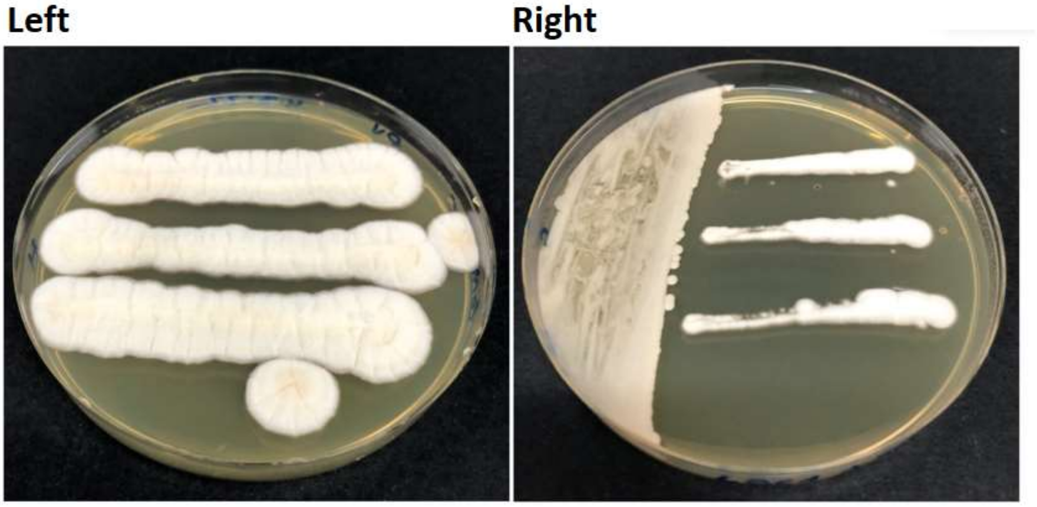

Figure S1. Representative photograph of the streak inhibition assay. The image on the left shows the growth of *Penicillium verrucosum* PV21 in YPDA medium without yeast and, on the right, streaks of the same fungus grown with yeast (LRC1).

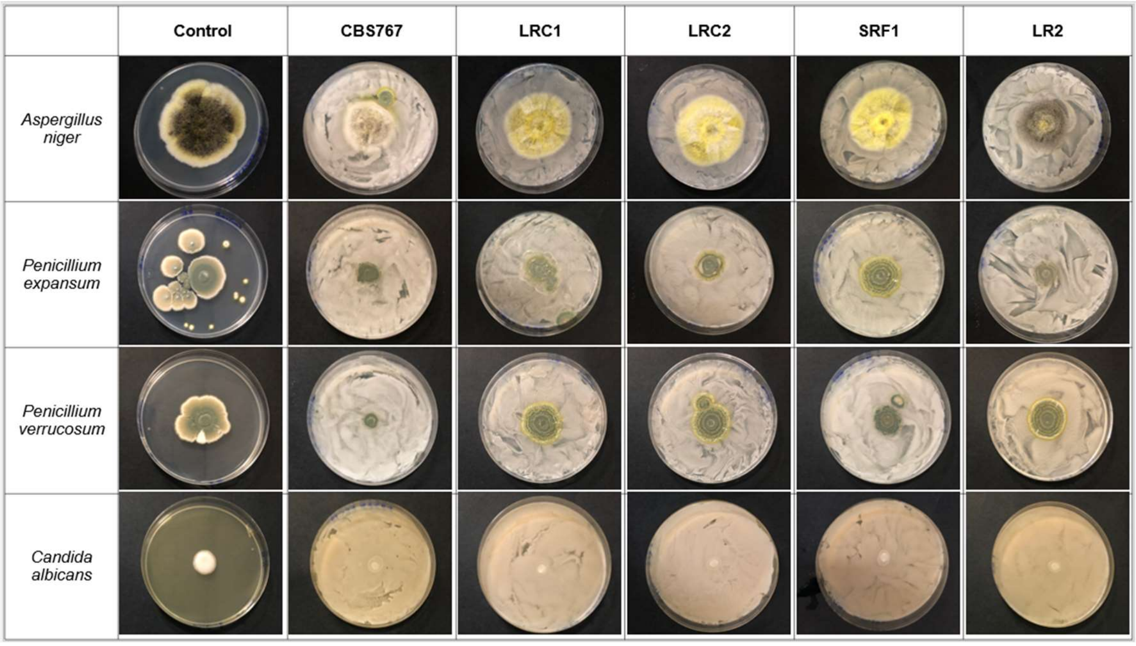

Figure S2. A) Representative photographs of the test of radial inhibition of potentially pathogenic fungi by different strains of *Debaryomyces hansenii*. From left to right the different strains used and, from top to bottom, the potentially pathogenic fungi under study.

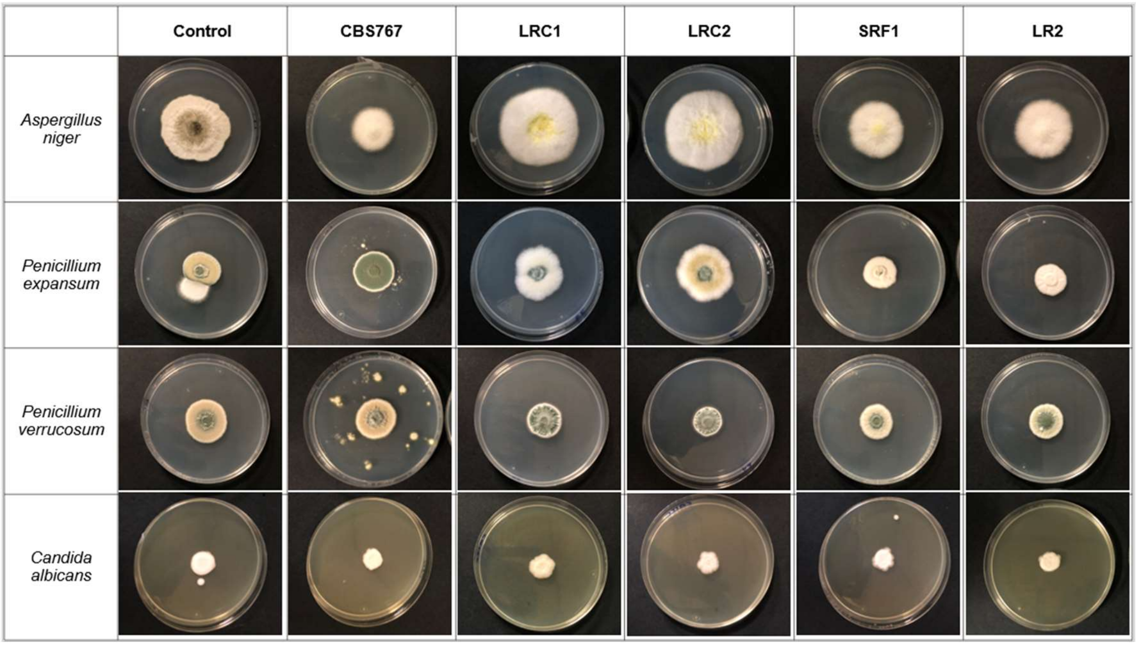

Figure S3. Representative photographs of the inhibition test for volatile compounds produced by *Debaryomyces hansenii* against potential pathogenic fungi. From left to right the different strains used and, from top to bottom, the potentially pathogenic fungi under study.

Table S1. MALDI Biotyper identification results.

Yeasts Strains	Score Value	Matched Pattern	NCBI Identifier
CBS767	2.20	*Debaryomyces hansenii*	4959
LRC1	2.06	*Debaryomyces hansenii*	4959
LRC2	2.03	*Debaryomyces hansenii*	4959
SRF1	2.03	*Debaryomyces hansenii*	4959
LR2	2.12	*Debaryomyces hansenii*	4959

Meaning of score values:
0.00-1.69 No confidence microorganism identification.
1.70-1.99 Low-confidence microorganism identification.
2.00-3.00 High-confidence microorganism identification.

Table S2. Statistical analysis of the inhibitory potential of *Debaryomyces hansenii* strains. Comparison of inhibitory potential of each strain against each other for every NaCl concentration studied (0, 0.5, and 1 M). The inhibitory effect of the different *D. hansenii* strains was determined by comparing maximum mycelial growth in control (without yeast) and cocultured plates.

**Comparisons**	*Aspergillus niger*			*Penicillium expansum*			*Penicillium verrucosum*			*Candida albicans*		
	0 M	0.5 M	1 M	0 M	0.5 M	1 M	0 M	0.5 M	1 M	0 M	0.5 M	1 M
CBS767 *vs.* LRC1	ns	*	ns	ns	ns	ns	ns	ns	ns	*	ns	ns
CBS767 *vs.* LRC2	***	ns	ns	ns	**	ns	**	ns	**	***	ns	ns
CBS767 *vs.* SRF1	*	ns	ns	ns	*	ns	ns	ns	ns	ns	ns	ns
CBS767 *vs.* LR2	*	*	ns	ns	ns	*	**	ns	*	*	ns	ns
LRC1 *vs.* LRC2	***	ns	ns	ns	ns	**	***	ns	**	***	ns	ns
LRC1 *vs.* SRF1	***	ns	ns	ns	ns	ns	ns	ns	ns	ns	ns	ns
LRC1 *vs.* LR2	***	ns	ns	ns	ns	ns	**	ns	*	ns	ns	ns
LRC2 *vs.* SRF1	*	ns	ns	ns	ns	ns	ns	ns	**	***	ns	ns
LRC2 *vs.* LR2	*	ns	ns	ns	ns	**	ns	ns	ns	**	ns	ns
SRF1 *vs.* LR2	ns	ns	ns	ns	ns	**	ns	ns	ns	ns	ns	ns

ns: not significant, * $p < 0.05$, ** $p < 0.01$, *** $p < 0.001$

Table S3. Statistical analysis of the effect of different NaCl concentrations on the inhibitory potential of each *Debaryomyces hansenii* strain. A comparison of inhibitory potential between NaCl concentrations studied (0, 0.5, and 1 M) for each *D. hansenii* strain. The inhibitory effect of the different *D. hansenii* strains was determined by comparing maximum mycelial growth in control (without yeast) and cocultured plates.

**Strains**	**Conditions (NaCl)**	***Aspergillus niger***	***Penicillium expansum***	***Penicillium verrucosum***	***Candida albicans***
CBS767	0 *vs.* 0.5 M	*	**	*	ns
	0 *vs.* 1 M	ns	ns	ns	ns
	0.5 M *vs.* 1 M	*	**	*	ns
LRC1	0 *vs.* 0.5 M	*	***	**	***
	0 *vs.* 1 M	*	ns	ns	***
	0.5 M *vs.* 1 M	ns	**	**	***
LRC2	0 *vs.* 0.5 M	ns	ns	ns	**
	0 *vs.* 1 M	ns	**	*	***
	0.5 M *vs.* 1 M	ns	**	*	*
SRF1	0 *vs.* 0.5 M	ns	**	ns	*
	0 *vs.* 1 M	ns	**	ns	ns
	0.5 M *vs.* 1 M	ns	***	ns	**
LR2	0 *vs.* 0.5 M	ns	*	ns	ns
	0 *vs.* 1 M	ns	ns	***	ns
	0.5 M *vs.* 1 M	ns	ns	**	ns

ns: not significant, * $p < 0.05$, ** $p < 0.01$, *** $p < 0.001$

Table S4. Statistical analysis of the radial growth assay of unwanted fungi cocultured with *Debaryomyces hansenii*. A comparison between the inhibitory activity of each strain against the control plates (without *D. hansenii*) and each other. The inhibitory activity of the different *D. hansenii* (not shown) was measured as described in the manuscript (IA). The control plates were considered to have an inhibitory activity of 0%.

**Comparisons**	*Aspergillus niger*	*Penicillium expansum*	*Penicillium verrucosum*	*Candida albicans*
Control *vs.* CBS767	***	***	***	***
Control *vs.* LRC1	*	***	ns	***
Control *vs.* LRC2	ns	***	ns	***
Control *vs.* SRF1	*	***	*	***
Control *vs.* LR2	**	***	ns	***
CBS767 *vs.* LRC1	*	ns	*	ns
CBS767 *vs.* LRC2	**	ns	**	ns
CBS767 *vs.* SRF1	*	*	*	ns
CBS767 *vs.* LR2	ns	ns	**	ns
LRC1 *vs.* LRC2	ns	ns	ns	ns
LRC1 *vs.* SRF1	ns	ns	ns	ns
LRC1 *vs.* LR2	*	ns	ns	ns
LRC2 *vs.* SRF1	ns	ns	ns	ns
LRC2 *vs.* LR2	*	ns	ns	ns
SRF1 *vs.* LR2	ns	*	ns	ns

Control: Growth of the unwanted fungi in the absence of any *D. hansenii* strain. ns: not significant, * $p < 0.05$, ** $p < 0.01$, *** $p < 0.001$
